# Metformin and Risks of Aortic Aneurysm and Aortic Dissection: A Mendelian Randomization Study

**DOI:** 10.31083/RCM27734

**Published:** 2025-04-27

**Authors:** Lei Wang, Ziyan Lin, Yuzuo Lin, Qingtong Wu, Guodong Zhong, Liangwan Chen

**Affiliations:** ^1^Department of Cardiovascular Surgery, Fujian Medical University Union Hospital, 350000 Fuzhou, Fujian, China; ^2^Key Laboratory of Cardio-Thoracic Surgery (Fujian Medical University), Fujian Province University, 350000 Fuzhou, Fujian, China; ^3^Union College of Clinical Medicine, Fujian Medical University, 350000 Fuzhou, Fujian, China; ^4^Department of Pathology, Fujian Province Second People’s Hospital: The Second Affiliated Hospital of Fujian University of Traditional Chinese Medicine, 350000 Fuzhou, Fujian, China; ^5^Engineering Research Center of Tissue and Organ Regeneration, Fujian Province University, 350000 Fuzhou, Fujian, China

**Keywords:** metformin, aortic aneurysm, aortic dissection, Mendelian randomization

## Abstract

**Background:**

Previous research has suggested that metformin may inhibit the dilation of an abdominal aortic aneurysm (AAA); however, these findings are controversial. Additionally, limited reporting exists on the relationships between metformin and thoracic aortic aneurysm (TAA) and aortic dissection (AD). Therefore, this study aimed to assess the potential relationship between metformin and the risk of aortic aneurysm (AA)/AD using the Mendelian randomization (MR) analysis.

**Methods:**

Genome-wide association studies and FinnGen summary data were utilized for the MR analysis. The causal relationship between metformin and AA/AD was primarily assessed using the inverse-variance weighted (IVW) method. Sensitivity analyses were conducted to detect heterogeneity and pleiotropy.

**Results:**

The results indicated a negative correlation between metformin treatment and the risk of both AA and AD, with odds ratios(ORs) reported as follows: OR = 0.010, 95% confidence interval (CI):0.000–0.212, *p* = 0.003 for AA, OR = 0.004, 95% CI: 0.000–0.220, *p* = 0.007 for abdominal aortic aneurysm (AAA); OR = 0.017, 95% CI: 0.000–0.815, *p* = 0.039 for thoracic aortic aneurysm (TAA); and OR = 0.001, 95% CI: 0.000–0.531, *p* = 0.032 for AD using the IVW method. These findings suggested that metformin might act as a protective factor against the occurrence of AA/AD. Furthermore, sensitivity analyses validated the robustness of these findings.

**Conclusions:**

This MR analysis identified a potential genetic causal relationship between metformin use and the risks of AA/AD, suggesting that metformin could serve as a protective agent in decreasing the incidences of these conditions.

## 1. Introduction

Aortic diseases include aortic aneurysms and acute aortic dissection (AD). Aortic aneurysm (AA) ranks as the second most prevalent aortic disease, following atherosclerosis [1]. It is defined as an abnormal dilation of the aortic walls, which includes thoracic aortic aneurysm (TAA) and abdominal aortic aneurysm (AAA). The risk of mortality due to aneurysm rupture is exceedingly high; patients with AAA face a mortality rate ranging from 60–70%, leading to approximately 150,000 to 200,000 deaths annually attributed to AAA rupture [2]. Acute AD is considered a critical cardiovascular condition associated with elevated rates of mortality and morbidity, often necessitating urgent surgical intervention. This condition arises from the rupture in the intima layer of the aorta, permitting blood to penetrate into the middle layer of the arterial wall and resulting in the formation of a dissection hematoma. If left untreated, most patients succumb within hours or days following the onset of acute AD. The global mortality rates associated with aortic diseases are increasing [2]. These conditions are linked to several risk factors, including smoking, hypertension, inflammation, dyslipidemia, infections and genetic variations; however, their precise mechanisms remain incompletely understood. Currently available treatment modalities for AA and AD encompass open surgical procedure, hybridization techniques, total endoluminal repair, and pharmacological therapy. Among these options, operative intervention remains the primary therapeutic approach. However, the elevated surgical risks and postoperative morbidity impose significant burdens on healthcare systems. Furthermore, the diameters of AAAs identified were predominantly below the threshold for operative intervention, often discovered through screening high-risk populations [3] or incidentally during abdominal imaging examinations, making adoption of pharmacologic therapy crucial in limiting AA progression. At present, there is insufficient evidence to support the effectiveness of medications such as doxycycline and angiotensin-converting enzyme inhibitors in preventing AAA dilation [4, 5]. Therefore, it is imperative to identify a safe, effective, and affordable therapeutic agent for inhibiting AA/AD, which represents an urgent public health concern.

Metformin, recognized as a first-line pharmacotherapy for type 2 diabetes, has demonstrated efficacy in regulating blood glucose levels through the inhibition of hepatic glucose production, enhancement of peripheral insulin sensitivity, and improvement in glucose uptake and utilization. It is well-regarded for its high safety profile and tolerability. Furthermore, metformin has been associated with benefits in promoting weight loss and metabolic improvement among obese adolescents [6]. Clinical studies suggest that metformin may have the potential to reduce cardiovascular events in diabetic patients; this effect may be attributed to its influence on weight reduction [7, 8]. An animal study has also indicated that metformin can inhibit the development and progression of AAA by preserving medial elastin and smooth muscle, as well as reducing infiltration levels of macrophages and CD8+ T cells within the aortic wall [9]. Systematic reviews and meta-analyses have further demonstrated that metformin may restrict AAA expansion and mitigate events associated with AAA [10, 11]. Consequently, metformin shows promise as a potential agent for the prevention or treatment of AA/AD. However, a study has shown that metformin does not alleviate inflammation in diabetic patients with AAA or those at elevated risk of AAA formation [12]. Vignac *et al*. [13] discovered that metformin therapy does not correlate with a reduced prevalence of ascending aortic aneurysm in individuals with diabetes. Moreover, the effects of metformin on AAA have yet to be validated through randomized controlled trials (RCTs), and there is a paucity of research examining the relationship between metformin use and the risks associated with TAA or AD. Additionally, various confounding factors—including diabetes mellitus status, utilization of oral hypoglycemic agents, hypertension, dyslipidemia, and obesity—may skew the outcomes of clinical studies, leading to potential biases. Consequently, further epidemiological investigations are essential to eliminate these confounding variables and more accurately assess the causal relationships between metformin and risks of AA/AD.

Mendelian randomization (MR) is a unique epidemiological research methodology grounded in Mendelian laws of heredity [14]. This approach utilizes single-nucleotide polymorphisms (SNPs) that demonstrate a significant association with exposure as instrumental variables (IVs), thereby elucidating potential causal relationships between exposures and outcomes. By doing so, MR minimizes confounding factors and mitigates the risk of reverse causation bias [14, 15]. In scenarios where RCTs are impractical due to ethical considerations or limited funding, evidence derived from MR analysis may provide a higher level of support for causal inference. Given that specific SNP alleles are randomly allocated during meiosis of germ cells, genetic variation remains unaffected by potential confounders [16]. As a result, MR analysis effectively reduces confounding influences and circumvents reverse causality, significantly enhancing the reliability of findings. Therefore, two-sample MR analysis was utilized in this study to evaluate the causal relationship between metformin and AA/AD.

## 2. Materials and Methods

### 2.1 Research Design

**Fig. 1.**
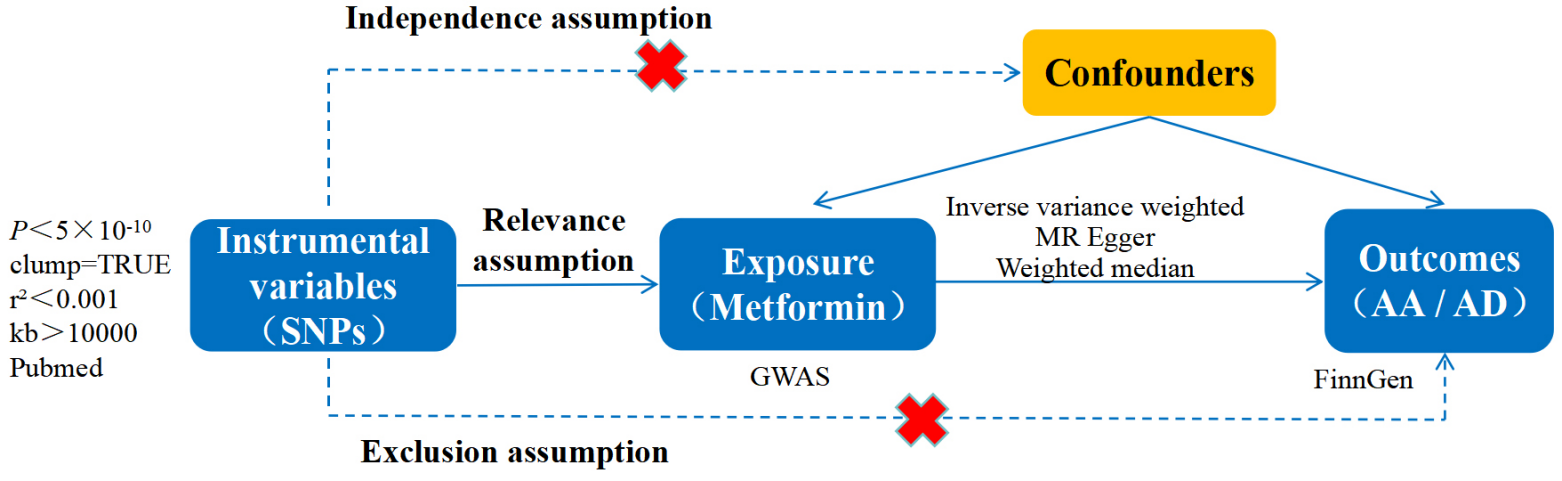
**The thorough design of the current Mendelian randomization analysis**. SNPs, single nucleotide polymorphisms; GWAS, genome-wide association studies; AA, aortic aneurysm; AD, aortic dissection; MR, Mendelian randomization.

A two-sample MR analysis was performed to explore the causal relationship between metformin and AA/AD. The exposure variable under investigation was metformin, while the IVs were SNPs that exhibited a significant association with metformin. The outcome variables were AA/AD. Three fundamental assumptions of MR analysis [15] are as follows: (1) Relevance assumption: The IVs demonstrate a stable correlation with the exposure variable, which in this case is metformin. (2) Exclusion assumption: The IVs influence the incidence of AA/AD solely through their effect on metformin, without involving any alternative pathways. (3) Independence assumption: There are no confounding factors affecting both the IVs and AA/AD. The flowchart illustrating the MR analysis was presented in Fig. 1.

### 2.2 Data Sources

The Genome-Wide Association Study (GWAS) dataset pertaining to metformin treatment was sourced from the GWAS database, while datasets for AA, AAA, TAA, and AD were obtained from the FinnGen database. The baseline characteristics of both the exposure variable and outcome variables were presented in Table 1 (Ref. [17]). The diagnostic criteria for outcome variables were outlined in **Supplementary Table 1**. Both the GWAS and FinnGen datasets are publicly accessible and have received approval from the relevant ethical committee. As a result, no additional ethical approval was necessary for the analyses conducted in this study.

**Table 1.** **Baseline characteristics of metformin treatment, AA, AAA, TAA, and AD datasets**.

	Trait	ID	Year	Sample size	n Case	n Control	Population	n SNP
Exposure variable	Metformin treatment	GWAS	2017	337,159	8392	328,767	European	10,894,596
		ukb-a-159						
Outcome variables	AA	finngen_R10_I9_	2023	390,102	8125	381,977	European	19,682,397
		AORTANEUR [17]						
	AAA	finngen_R10_I9_	2023	385,846	3869	381,977	European	19,682,330
		ABAORTANEUR [17]						
	TAA	finngen_R10_I9_	2023	385,857	3880	381,977	European	19,682,294
		THAORTANEUR [17]						
	AD	finngen_R10_I9_	2023	289,318	967	381,977	European	19,682,352
		AORTDIS [17]						

AA, aortic aneurysm; AAA, abdominal aortic aneurysm; TAA, thoracic aortic aneurysm; AD, aortic dissection; GWAS, genome-wide association studies; SNP, single nucleotide polymorphism.

### 2.3 Selection of Instrumental Variables

SNPs that exhibited a significant association with metformin (*p* < 5 × 10^-10^) were identified as IVs. To eliminate SNPs exhibiting linkage disequilibrium, thresholds of R^2^ < 0.001 and kb > 10,000 were established. Additionally, palindromic SNPs were excluded to ensure that the effects of these SNPs on the exposure variable corresponded to the same allele as their effect on the outcome variables. Furthermore, F statistics were utilized to assess potential weak IV bias. The F statistic was calculated using the formula F = R^2^ (N-K-1)/[K (1-R^2^)], where R^2^ denotes the cumulative explained variance attributed to the SNPs during exposure, N represents the sample size of the exposure dataset, and K indicates the number of SNPs included in the final analysis. A strong predictive power for SNPs on the exposure variable was indicated by F-statistics >10; therefore, SNPs exhibiting an F statistic <10 were excluded from subsequent analyses. Subsequently, we further excluded SNPs associated with confounding factors related to outcome variables—such as hypertension, hyperlipidemia, and smoking—by consulting resources available on PubMed.

### 2.4 Two-Sample MR Analysis

The causal relationship between metformin and AA/AD was assessed using five MR analysis methods: inverse variance weighted (IVW) as the primary method, supplemented by MR Egger, weighted median, weighted mode, and simple mode methods. The MR results were visualized utilizing the “TwoSampleMR” R package, which facilitated the generation of scatter plots, forest plots, and funnel plots, with a particular emphasis on the IVW findings. Scatter plots exhibiting a minimal intercept suggest that confounding factors have little impact on the reliability of the results. A positive slope in these plots indicates that the exposure variable act as a risk factor, whereas a negative slope signifies it serves as a protective factor. Forest plots were used to evaluate the predictive efficacy of each SNP concerning the outcome variables. The efficacy value was denoted by the β value, along with its range of variability. The β value and the odds ratio (OR) value can be interconnected through an exponential transformation. Solid dots positioned on the left indicate protective factors associated with SNPs, while solid dots on the right denote risk factors. Funnel plots were used to evaluate randomization quality. A symmetrical distribution of IVs around both side of the IVW line would indicate compliance with Mendel’s second law regarding random grouping. A *p* value less than 0.05 was considered indicative of a statistically significant causal relationship between exposure and outcomes.

### 2.5 Sensitivity Analysis

Sensitivity analyses were performed to evaluate the robustness of the MR findings. The heterogeneity of SNPs in both the IVW and MR Egger methods was conducted utilizing Cochran’s Q test, with a *p* value greater than 0.05 indicating no significant heterogeneity among the selected IVs. The presence of horizontal pleiotropy among SNPs was investigated through the MR Egger intercept and MR-PRESSO methods, where a *p* value exceeding 0.05 suggested an absence of horizontal pleiotropy, thereby indicating no confounding factors within the study. Additionally, a “leave-one-out” method was employed to reassess the effect estimates of the remaining SNPs after individually excluding each SNP; any notable changes in effect values signified potential significant impacts on causal relationships, warranting their removal from further analysis. The efficacy value was represented by the β value, along with its variability range illustrated in the forest plots and “leave-one-out” forest plots.

### 2.6 Statistical Analysis

The data were analyzed using the “TwoSampleMR” and “MR-PRESSO” packages within R software version 4.3.3 (The R Foundation for Statistical Computing, Vienna, Austria).

## 3. Results

### 3.1 Selection of SNPs

After excluding SNPs that demonstrated linkage disequilibrium, palindromic SNPs, those with F-statistics below 10, and SNPs associated with confounding factors, a total of 15 SNPs related to metformin were included in the MR analysis.

### 3.2 The Causal Relationship between Metformin and AA, AAA, TAA, and AD

**Fig. 2.**
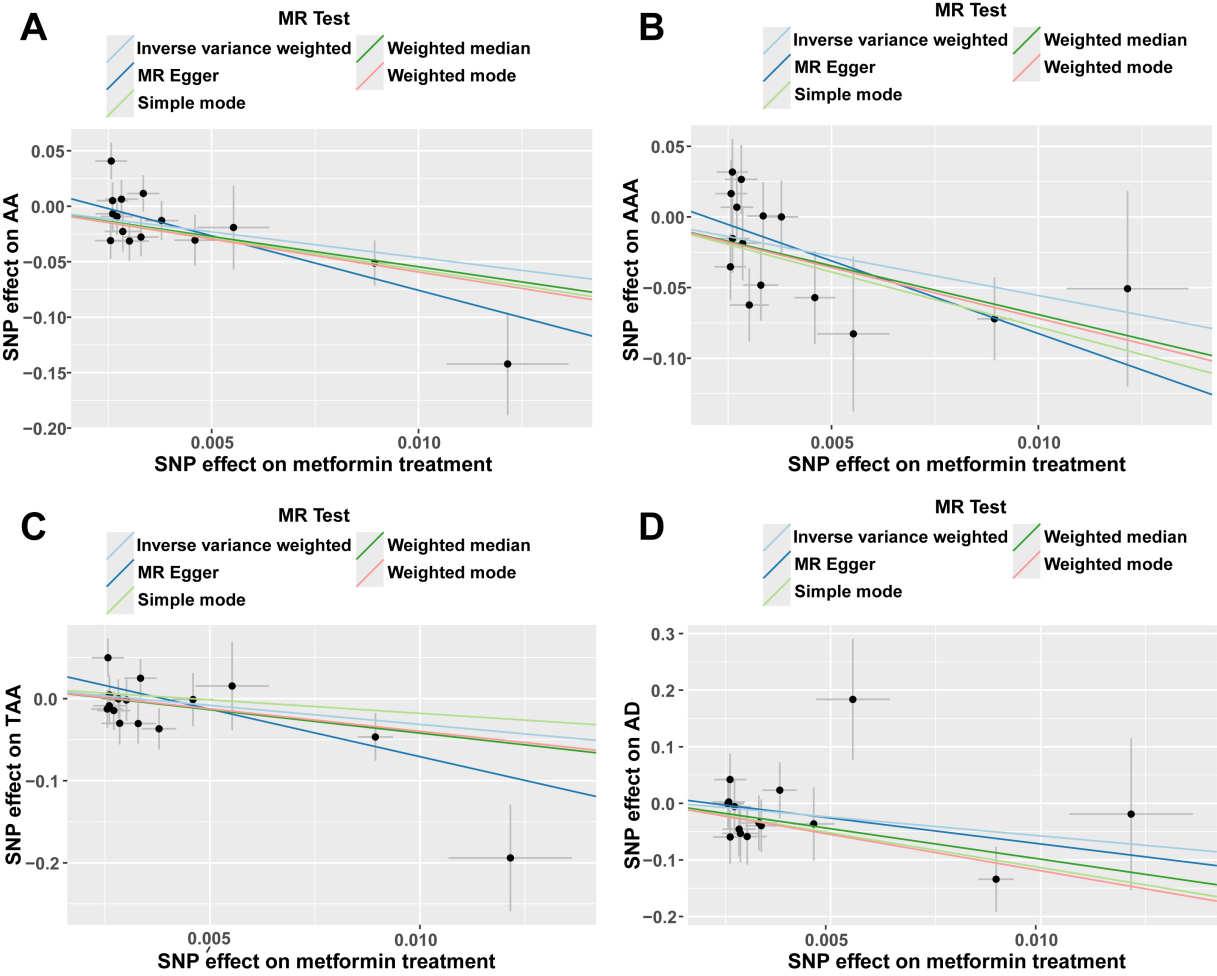
**Scatter plots of MR analysis, primarily using the IVW method**. (A) Scatter plot illustrating the MR analysis between metformin and AA. (B) Scatter plot depicting the MR analysis of metformin in relation to AAA. (C) Scatter plot representing the MR analysis of metformin concerning TAA. (D) Scatter plot showcasing the MR analysis of metformin with respect to AD. AA, aortic aneurysm; AAA, abdominal aortic aneurysm; TAA, thoracic aortic aneurysm; AD, aortic dissection; IVW, inverse variance weighted; MR, Mendelian randomization; SNP, single nucleotide polymorphism.

**Fig. 3.**
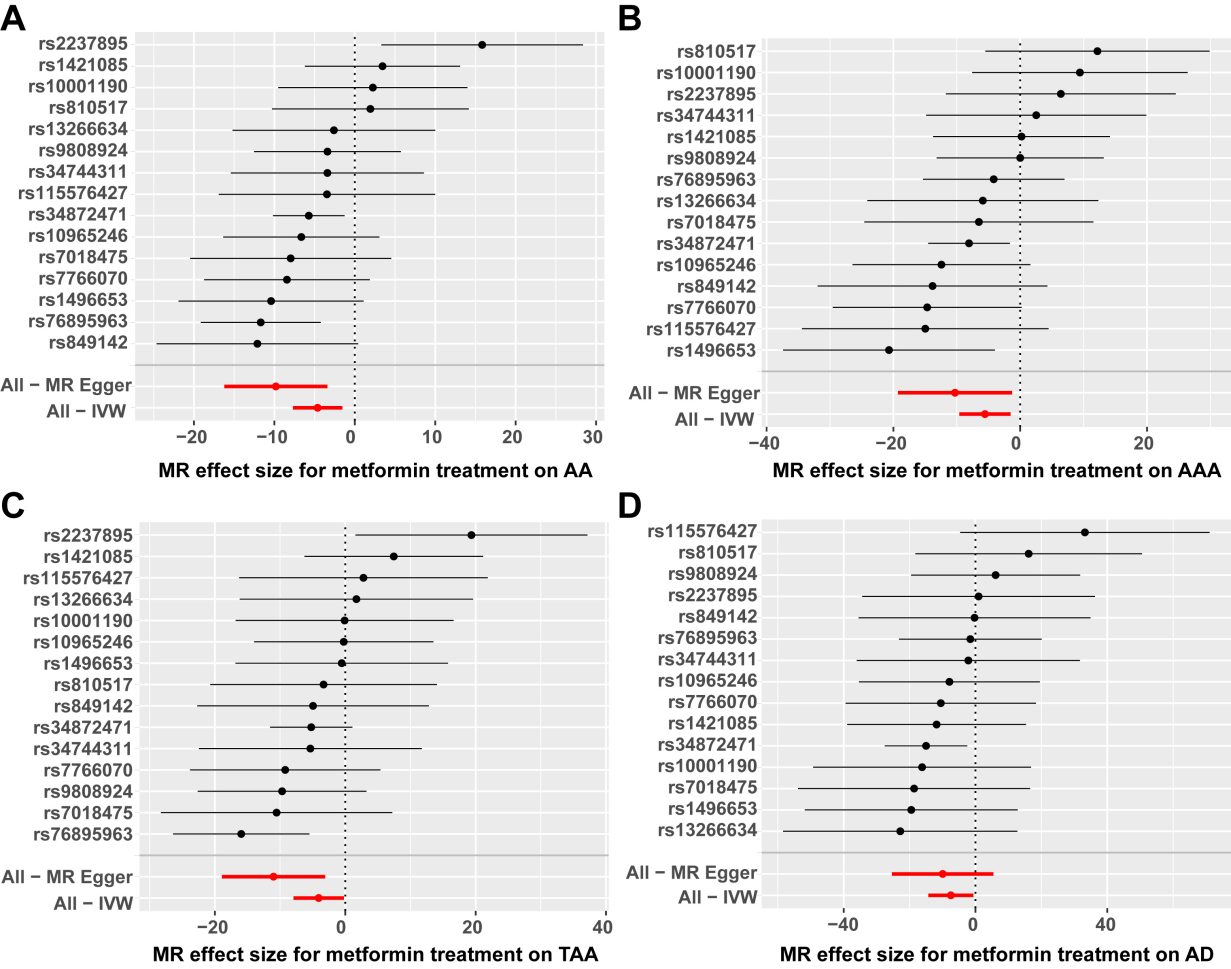
**Forest plots of MR analysis, primarily using the IVW method**. (A) Forest plot of MR analysis between metformin and AA. (B) Forest plot of MR analysis between metformin and AAA. (C) Forest plot of MR analysis between metformin and TAA. (D) Forest plot of MR analysis between metformin and AD. AA, aortic aneurysm; AAA, abdominal aortic aneurysm; TAA, thoracic aortic aneurysm; AD, aortic dissection; IVW, inverse variance weighted; MR, Mendelian randomization.

**Fig. 4.**
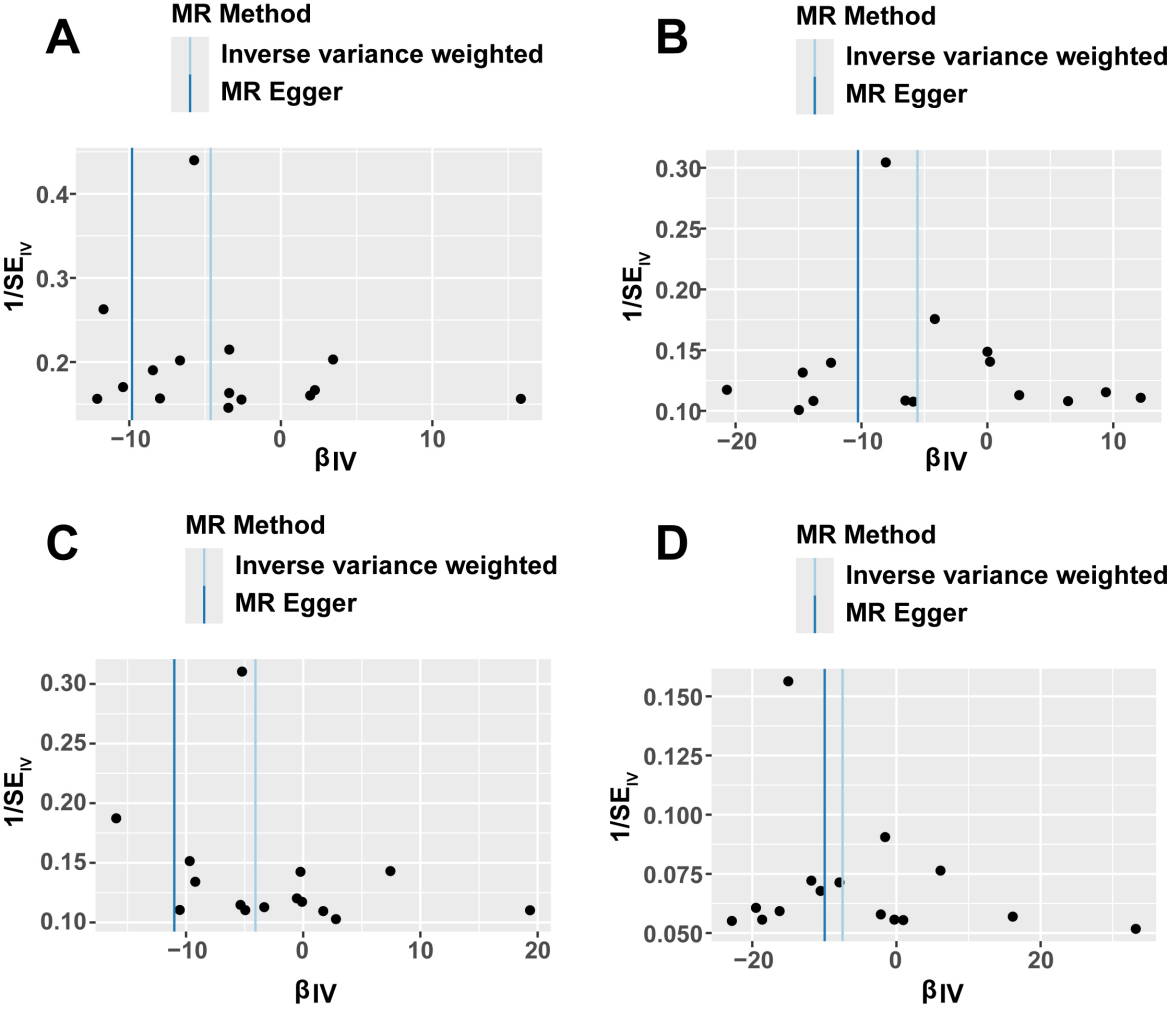
**Funnel plots of MR analyses, primarily using the IVW method**. (A) Funnel plot of MR analysis of metformin and AA. (B) Funnel plot of MR analysis of metformin and AAA. (C) Funnel plot of MR analysis of metformin and TAA. (D) Funnel plot of MR analysis of metformin and AD. AA, aortic aneurysm; AAA, abdominal aortic aneurysm; TAA, thoracic aortic aneurysm; AD, aortic dissection; IVW, inverse variance weighted; MR, Mendelian randomization; IV, instrumental variable; SE, standard error.

The IVW method demonstrated a causal association between metformin and AA, AAA, TAA, and AD, identifying metformin as a protective factor in all instances. This finding suggested that metformin might exert a preventive effect on the occurrence of AA, AAA, TAA, and AD (Table 2). The consistency of these associations was corroborated through MR Egger, weighted median, and most weighted mode methods, underscoring the robustness of the results (**Supplementary Table 2**, **Supplementary Fig. 1**). Scatter plots, forest plots and funnel plots illustrating the MR analysis of metformin and AA, AAA, TAA and AD were presented in Figs. 2,3,4, respectively. Among the four aforementioned causal associations, the small intercept observed in the scatter plot indicated minimal influence from confounding factors on both exposure and outcome variables; this enhanced the reliability of the results. Additionally, the negative slope of the line indicated that metformin acted as a protective factor against AA, AAA, TAA, and AD. The total predictive efficacy values of SNPs concerning outcome variables depicted in the forest plot were located on left side—further supporting that metformin served as a protective agent. Additionally, the symmetry observed in SNP distribution within funnel plots suggested relative stability of these results.

**Table 2.** **MR analysis of exposure and outcome variables using the IVW method and the sensitivity analysis**.

Exposure variable	Outcome variables	n SNP	OR (95% CI), IVW	*p* value, IVW	Heterogeneity test. *p*		Pleiotropy test. *p*	
					IVW	MR-Egger.intercept	MR-Egger.intercept	MR.PRESSO.Global. test
Metformin treatment	AA	15	0.010 (0.000–0.212)	0.003	0.068	0.152	0.099	0.081
Metformin treatment	AAA	15	0.004 (0.000–0.220)	0.007	0.183	0.206	0.272	0.199
Metformin treatment	TAA	15	0.017 (0.000–0.815)	0.039	0.216	0.383	0.077	0.233
Metformin treatment	AD	15	0.001 (0.000–0.531)	0.032	0.645	0.578	0.730	0.615

MR, Mendelian randomization; IVW, inverse variance weighted; SNP, single nucleotide polymorphism; OR, odds ratio; CI, confidence interval; AA, aortic aneurysm; AAA, abdominal aortic aneurysm; TAA, thoracic aortic aneurysm; AD, aortic dissection.

### 3.3 Sensitivity Analysis

**Fig. 5.**
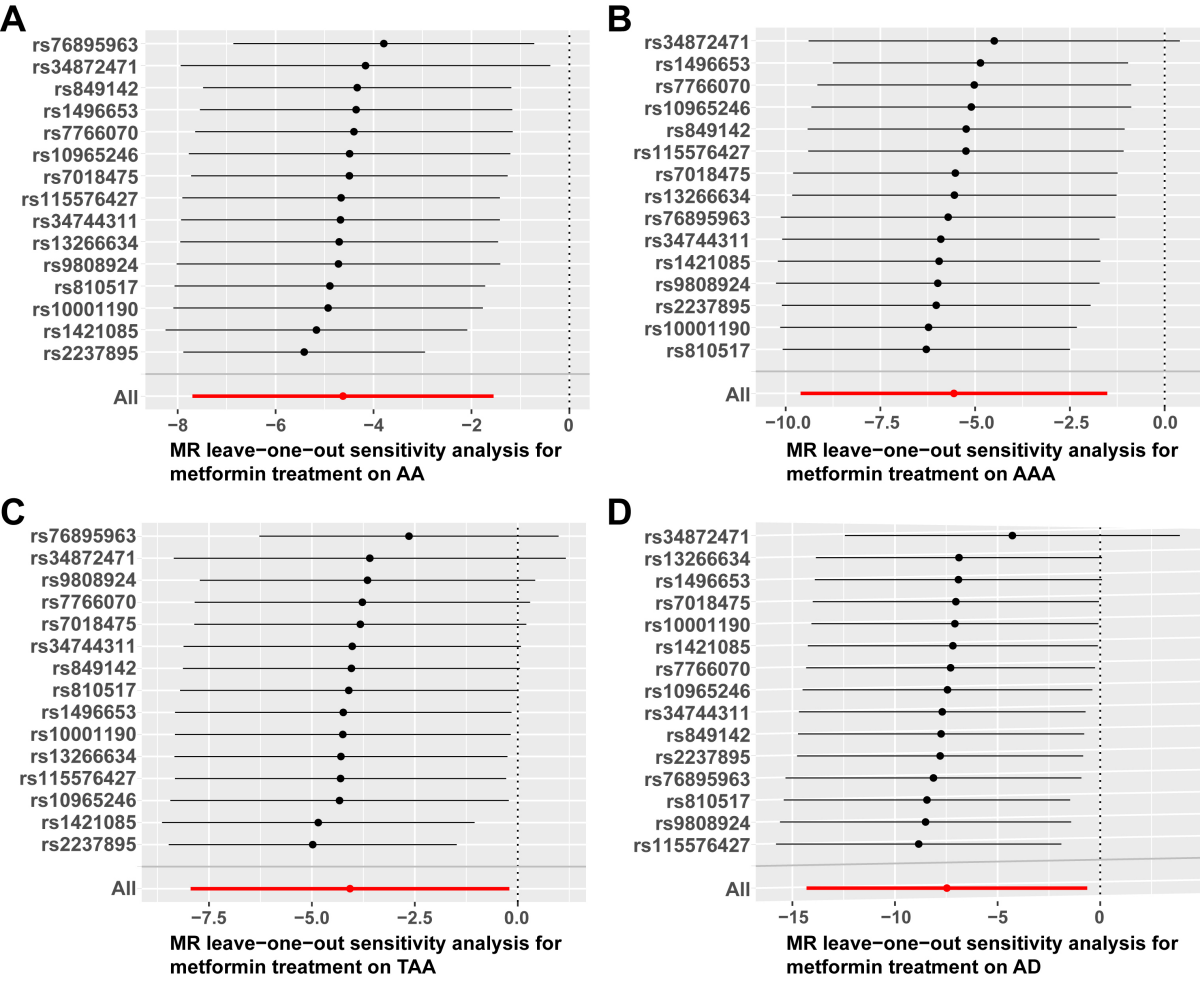
**Forest plots of the “leave-one-out” method for MR analysis**. (A) Forest plot depicting the “leave-one-out” method of metformin and AA. (B) Forest plot representing the “leave-one-out” MR method of metformin and AAA. (C) Forest plot showcasing the “leave-one-out” method of metformin and TAA. (D) Forest plot of “leave-one-out” method of metformin and AD. AA, aortic aneurysm; AAA, abdominal aortic aneurysm; TAA, thoracic aortic aneurysm; AD, aortic dissection; MR, Mendelian randomization.

Heterogeneity was evaluated using the IVW and MR Egger methods. The *p* values for the selected SNPs were all greater than 0.05, indicating a lack of heterogeneity. To assess the presence of horizontal pleiotropy among the SNPs, we employed both MR Egger intercept and MR-PRESSO analyses. The results revealed that all *p* values exceeded 0.05, suggesting no evidence of pleiotropy in the SNPs (Table 2). The “leave-one-out” analysis demonstrated that when any SNP was removed, the entire error bar remained on one side of the IVW line, indicating that each SNP exerted an equal effect on the results without significant interference from any individual SNP (Fig. 5). These findings enhance the reliability of our study outcomes.

## 4. Discussion

In this study, we conducted a two-sample MR analysis to explore the potential causal relationship between metformin and AA/AD. The results revealed a causal relationship between metformin use and the risk of AA/AD, indicating that metformin functions as an upstream protective factor against AA/AD. This suggests that metformin may be beneficial in reducing the incidence of AA/AD and in slowing down disease progression. For patients with AA/AD who are not suitable candidates for surgical intervention, effective pharmacotherapy can alleviate patient concerns and enhance overall prognosis.

Diabetes is a recognized risk factor for cardiovascular disease. However, numerous studies—including epidemiological research, animal experiments, and meta-analyses—have indicated that diabetes is associated with a lower incidence of AAA and a slower progression of the disease [18, 19, 20]. The precise mechanism by which diabetes influences the aorta remain unclear; however, potential factors may include impaired vascular generation, abnormal interactions between monocytes and the extracellular matrix, increased collagen cross-linking resulting from the accumulation of advanced glycation end products, dysregulation of cell cycle proteins, and off-target effects from medications used in diabetes management [21]. Additionally, the administration of metformin for diabetes management may also have significant implications. For instance, Golledge *et al*. [22] found that diabetic patients who were prescribed metformin exhibit a lower incidence of AAA events compared to their non-diabetic counterparts. In contrast, diabetic patients who did not receive metformin did not demonstrate this reduced incidence [22]. Besides, a cohort study conducted in Sweden revealed that metformin among individuals with type 2 diabetic patients is associated with a decreased growth rate of AAA and lower levels of chemokine expression [23]. This effect may be attributed to the anti-inflammatory properties inherent in metformin. However, further investigation is warranted to elucidate the impact of metformin on AAA growth rates in non-diabetic patients.

Metformin not only exhibits glycemic control properties but also demonstrates vasoprotective effects. Han *et al*. [24] demonstrated that metformin is effective in reducing cardiovascular mortality, all-cause mortality, and the incidence of cardiovascular events among patients with coronary artery disease. Furthermore, metformin has been found to enhance endothelial function in both rodent models and human subjects [25, 26], improving endothelium-dependent relaxations (EDRs) and alleviating endothelial dysfunction associated with hyperglycemia and obesity in isolated mouse aortas through the activation of adenosine monophosphate (AMP)-activated protein kinase (AMPK) and inhibition of inflammation linked to the Yes-associated protein-c-Jun N-terminal kinase (YAP-JNK) pathway [27]. An animal study demonstrated that swimming combined with metformin can protect the hearts and aortas of obese type 2 diabetic rats from damage induced by high-fat diets via regulation of the B-cell lymphoma-2 (BCL2)/BCL2-associated X protein (Bcl2/Bax) signaling pathway [28]. Additionally, metformin also mitigates vascular calcification associated with hyperlipidemia by resisting ferroptosis in aortic smooth muscle cells [29]. Moreover, granuloma-targeted esculetin used alongside metformin improves age-related atherosclerosis by modulating AMPK activation [30] and reduces both atherogenesis and the progression of atherosclerosis in obese diabetic rats through modulation of the Sestrin2-mammalian target of rapamycin (mTOR) pathway [31]. These investigations into the impact of metformin on cardiovascular health provide additional support for this research’s findings.

For the aspect of aortic disease, an animal study demonstrated that metformin inhibits the proliferation of aortic smooth muscle cell and reduces the expression of matrix metalloproteinase 2 [9]. In murine experiments, metformin has been shown to impede early AAA progression by modulating AMPK activity, decreasing the production of interferon-gamma-expressing T cells, and enhancing the retention of circulating and splenic inflammatory monocytes [32]. Clinical studies and recent meta-analysis indicate that metformin significantly limits AAA expansion and may potentially reduce the risk of AAA-related events [10, 11]. Furthermore, it has been suggested that metformin may also lower mortality and morbidity associated with AAA repair surgery in diabetic patients [33]. Ma *et al*. [34] found that patient-derived microphysiological models effectively identify the therapeutic potential of metformin for treating TAA. Metformin may modulate both the contractile phenotype alterations and metabolic dysfunction in diseased human aortic smooth muscle cells, thereby limiting aortic dilation [34]. Although research on the protective mechanism by which metformin acts against AD is currently limited, its anti-inflammatory properties—alongside improvements in vascular endothelial cell function, enhanced performance of aortic smooth muscle cell, and weight loss—may contribute to reducing risks associated with atherosclerosis, hypertension, and incidence rates of AD. This MR analysis demonstrated that metformin not only provided protective effects against AAA but also against TAA and AD. This finding offered substantial evidence supporting ongoing research into the role of metformin in relation to aortic diseases.

In this MR study, the selection threshold for SNPs was established at *p* < 5 × 10^-10^, which was more stringent than the conventional threshold of *p* < 5 × 10^-8^. This rigorous criterion ensured a robust correlation between the IVs and the exposure factor while effectively mitigating potential biases arising from individual SNP variations on the study outcomes. The final number of SNPs included in this analysis was deemed sufficiently adequate to prevent significant statistical bias. The results obtained from the IVW, MR Egger, and weighted median method were consistent, with sensitivity analyses showing no significant heterogeneity or pleiotropy. Most confounding factors such as hypertension, diabetes mellitus and body weight were excluded, resulting in a more reliable causal relationship. However, it should be noted that neither the weighted mode nor simple mode methods all achieve statistical significance for causality. Furthermore, while this study demonstrated an absence of confounding through sensitivity analyses, it was important to acknowledge that assessing confounding factors is inherently complex; some unidentified confounding effects may still persist. Even if the exclusion assumption holds true, MR studies can be influenced by other limitations such as inadequate representation of genetic variation and data quality issues.

Observational studies examining the relationship between metformin and aortic disease can only establish associations between exposure and outcomes, rather than causality, and may be confounded by various factors. RCTs conducted globally are often costly and time-consuming; furthermore, the allocation of exposure factors poses ethical challenges due to the relatively low incidence of AA/AD. While RCTs investigating the effects of metformin on AAA are currently in progress [35], their results remain inconclusive. However, there is a notable absence of RCTs exploring the impact of metformin on TAA and AD. The findings from this MR study provide valuable supplementary evidence to existing observational research regarding the association between metformin use and aortic disease. Additionally, this study underscores the necessity for designing and implementing prospective studies. By integrating evidence from prospective research with our current findings, a more comprehensive analysis can be achieved.

When metformin is utilized in clinical practice, several factors must be considered. Firstly, a meta-regression analysis indicated that the relationship between metformin and AAA growth is significantly influenced by male gender. However, variables such as age, hypertension, diabetes, smoking history, and baseline diameter do not appear to exert a significant impact on this association [11]. Thus, the inhibitory effect of metformin on AAA growth may be more pronounced in males. Additionally, both smoking and the aging process may also influence this correlation [36]. Moreover, although concerns regarding potential side effects of metformin have been addressed through multiple randomized trials confirming its safety for individuals without diabetes [37], ongoing and meticulous monitoring of safety considerations in patients with aortic disease remains essential. Furthermore, TAA and AAA demonstrate distinct pathogenic mechanisms along with differing biomechanical and histological characteristics [38]. While AAA is primarily associated with hyperlipidemia and hypertension [39], TAA represents a more complex, multifactorial condition that lacks a clear correlation with diabetes. Lastly, it is important to note that this study was based on data from European populations. The response to metformin treatment may vary across different ethnic groups. Consequently, adjustments to dosage should be made considering the patient’s gender, age, race, medical history, and various types of aortic diseases.

There were some limitations in this study. Firstly, the current study is constrained by the lack of GWAS data on Asian and African populations, as it primarily focused on European cohorts. The exclusive use of European datasets restricts the generalizability of the findings to other demographic groups. Secondly, comprehensive data regarding the exposure variable—metformin treatment—was not accessible, which may introduce potential exposure bias. Thirdly, while we utilized MR to investigate the causal relationship between metformin and AA/AD, we did not extensively explore the underlying mechanisms within this study. Existing evidence does not elucidate how metformin specifically interacts with aortic pathology. Furthermore, findings from MR analysis should be integrated with prospective studies to achieve more robust evidence-based conclusions. Future research should adopt a holistic approach and incorporate multi-omics techniques to enhance our understanding of the intricate gene-disease-environment interactions that contribute to disease pathogenesis.

## 5. Conclusions

This study utilized a comprehensive genomic database derived from European populations and conducted a bivariate MR analysis to elucidate the potential causal relationship between metformin and AA/AD. The findings indicated that metformin is recognized as a protective agent in reducing the incidence of AA/AD. Various MR methodologies, along with sensitivity analyses, supported the relative reliability of these results; however, further prospective studies are necessary to validate the robustness of these findings.

## Data Availability

The data utilized in this manuscript was obtained from publicly accessible resources. The summary statistics for metformin treatment can be found at https://gwas.mrcieu.ac.uk/datasets/ukb-a-159/. Additionally, the summary data pertaining to aortic aneurysm, abdominal aortic aneurysm, thoracic aortic aneurysm, and aortic dissection are available at https://risteys.finngen.fi/endpoints/I9_AORTANEUR, https://r10.finngen.fi/pheno/I9_ABAORTANEUR, https://r10.finngen.fi/pheno/I9_THAORTANEUR, https://r10.finngen.fi/pheno/I9_AORTDIS,respectively.

## Abbreviations

AA, aortic aneurysm; AAA, abdominal aortic aneurysm; AD, aortic dissection; AMPK, adenosine monophosphate (AMP)-activated protein kinase; Bcl2/Bax, B-cell lymphoma-2 (BCL2)/BCL2-associated X protein; EDRs, endothelium-dependent relaxations; CI, confidence interval; GWAS, genome-wide association studies; IVs, instrumental variables; IVW, inverse variance weighted; MR, Mendelian randomization; mTOR, mammalian target of rapamycin; OR, odds ratios; RCTs, randomized controlled trials; SNPs, single-nucleotide polymorphisms; SE, standard error; TAA, thoracic aortic aneurysm; YAP-JNK, Yes-associated protein-c-Jun N-terminal kinase.

## Reporting Checklist

The authors have completed the STROBE reporting checklist.

## Supplementary Material

Supplementary material associated with this article can be found, in the online version, at https://doi.org/10.31083/RCM27734.
