# Supplementary material for: Metformin and Risks of Aortic Aneurysm and Aortic Dissection: A Mendelian Randomization Study
Source: Rev Cardiovasc Med. 2025 Apr 27;26(4):27734. doi: 10.31083/RCM27734 (PMC12059729; doi:10.31083/RCM27734)
Supplement: Supplementary file 1 [file 2153-8174-26-4-27734-s1.zip › Supplementary Material-V2.docx]

Supplementary Table S1 Diagnostic criteria of outcome variables

| Outcomes | ID | Diagnostic criteria |
| --- | --- | --- |
| AA | finngen_R10_I9_  AORTANEUR [16] | Aortic aneurysm was defined as a ruptured aneurysm located in the wall of the proximal portion of the descending aorta proceeding from the arch of the aorta.  International Classification of Diseases (ICD)-10  I71.1 Thoracic aortic aneurysm, ruptured  I71.2 Thoracic aortic aneurysm, without mention of rupture  I71.3 Abdominal aortic aneurysm, ruptured  I71.4 Abdominal aortic aneurysm, without mention of rupture  I71.5 Thoracoabdominal aortic aneurysm, ruptured  I71.6 Thoracoabdominal aortic aneurysm, without mention of rupture  I71.8 Aortic aneurysm of unspecified site, ruptured: Rupture of aorta  I71.9 Aortic aneurysm of unspecified site, without mention of rupture: Aneurysm of aorta, Dilatation of aorta, and Hyaline necrosis of aorta. |
| AAA | finngen_R10_I9_  ABAORTANEUR [16] | ICD-10  I71.3 Abdominal aortic aneurysm, ruptured  I71.4 Abdominal aortic aneurysm, without mention of rupture |
| TAA | finngen_R10_I9_  THAORTANEUR [16] | ICD-10  I71.01 Aneurysm of the descending aorta  I71.1 Thoracic aortic aneurysm, ruptured  I71.2 Thoracic aortic aneurysm, without mention of rupture  Q25.43 Aneurysm of sinus Valsalva, ruptured |
| AD | finngen_R10_I9_  AORTDIS [16] | ICD-10  I71.00 Dissecting aneurysm of aorta (ruptured) [any part]  I71.01 Aneurysm of the descending aorta  I71.09 Aortic dissection [regardless of location] |

AA, aortic aneurysm; AAA, abdominal aortic aneurysm; TAA, thoracic aortic aneurysm; AD, aortic dissection.

Supplementary Table S2 MR analysis between metformin and AA, TAA, AAA or AD utilizing five distinct MR methodologies

| Exposure | Outcomes | MR methods | n SNP | β | SE | β_lower_CI | β_upper_CI | β_pval | OR | OR_lower 95% CI | OR_upper_95% CI | OR_pval |
| --- | --- | --- | --- | --- | --- | --- | --- | --- | --- | --- | --- | --- |
| Metformin treatment | AA | MR Egger | 15 | -9.82 | 3.27 | -16.23 | -3.41 | 0.010 | 5.44E-05 | 8.93E-08 | 0.03 | 0.01 |
|  | AA | Weighted median | 15 | -5.46 | 1.81 | -9.00 | -1.92 | 0.003 | 4.27E-03 | 1.24E-04 | 0.15 | 0.002 |
|  | AA | Inverse variance weighted | 15 | -4.62 | 1.57 | -7.70 | -1.55 | 0.003 | 9.82E-03 | 4.54E-04 | 0.21 | 0.003 |
|  | AA | Simple mode | 15 | -5.73 | 3.00 | -11.60 | 0.14 | 0.077 | 3.25E-03 | 9.13E-06 | 1.15 | 0.079 |
|  | AA | Weighted mode | 15 | -5.93 | 2.22 | -10.28 | -1.57 | 0.018 | 2.66E-03 | 3.41E-05 | 0.21 | 0.015 |
|  | AAA | MR Egger | 15 | -10.28 | 4.60 | -19.29 | -1.28 | 0.043 | 3.42E-05 | 4.18E-09 | 0.28 | 0.043 |
|  | AAA | Weighted median | 15 | -6.91 | 2.53 | -11.88 | -1.95 | 0.006 | 9.96E-04 | 6.95E-06 | 0.14 | 0.009 |
|  | AAA | Inverse variance weighted | 15 | -5.56 | 2.06 | -9.61 | -1.51 | 0.007 | 3.85E-03 | 6.74E-05 | 0.22 | 0.007 |
|  | AAA | Simple mode | 15 | -7.79 | 4.71 | -17.02 | 1.43 | 0.120 | 4.12E-04 | 4.06E-08 | 4.20 | 0.158 |
|  | AAA | Weighted mode | 15 | -7.17 | 3.05 | -13.16 | -1.19 | 0.034 | 7.67E-04 | 1.93E-06 | 0.31 | 0.031 |
|  | TAA | MR Egger | 15 | -11.01 | 4.04 | -18.93 | -3.08 | 0.017 | 1.66E-05 | 6.00E-09 | 0.05 | 0.017 |
|  | TAA | Weighted median | 15 | -5.14 | 2.52 | -10.08 | -0.21 | 0.041 | 5.85E-03 | 4.20E-05 | 0.81 | 0.046 |
|  | TAA | Inverse variance weighted | 15 | -4.07 | 1.97 | -7.94 | -0.20 | 0.039 | 1.70E-02 | 3.55E-04 | 0.81 | 0.039 |
|  | TAA | Simple mode | 15 | -2.73 | 4.46 | -11.47 | 6.01 | 0.550 | 6.51E-02 | 1.04E-05 | 406.78 | 0.544 |
|  | TAA | Weighted mode | 15 | -4.92 | 3.16 | -11.12 | 1.28 | 0.142 | 7.32E-03 | 1.48E-05 | 3.61 | 0.116 |
|  | AD | MR Egger | 15 | -9.96 | 7.85 | -25.35 | 5.44 | 0.227 | 4.75E-05 | 9.79E-12 | 230.10 | 0.227 |
|  | AD | Weighted median | 15 | -11.58 | 4.55 | -20.50 | -2.65 | 0.011 | 9.38E-06 | 1.25E-09 | 0.07 | 0.015 |
|  | AD | Inverse variance weighted | 15 | -7.47 | 3.49 | -14.31 | -0.63 | 0.032 | 5.69E-04 | 6.10E-07 | 0.53 | 0.032 |
|  | AD | Simple mode | 15 | -13.07 | 8.50 | -29.73 | 3.60 | 0.147 | 2.11E-06 | 1.22E-13 | 36.53 | 0.122 |
|  | AD | Weighted mode | 15 | -13.62 | 5.84 | -25.06 | -2.18 | 0.035 | 1.21E-06 | 1.31E-11 | 0.11 | 0.034 |

AA, aortic aneurysm; TAA, thoracic aortic aneurysm; AAA, abdominal aortic aneurysm; AD, aortic dissection; MR, Mendelian randomization; SNP, single nucleotide polymorphism; SE, standard error; CI, confidence interval; OR, odds ratios.

Supplementary Fig. S1 MR analysis between metformin and AA, TAA, AAA or AD using methods of MR Egger, weighted median, inverse variance weighted, simple mode and weighted mode. AA, aortic aneurysm; TAA, thoracic aortic aneurysm; AAA, abdominal aortic aneurysm; AD, aortic dissection. SNP, single nucleotide polymorphism; OR, odds ratios; CI, confidence interval .
