# Supplementary figures and images for: Metformin and Risks of Aortic Aneurysm and Aortic Dissection: A Mendelian Randomization Study
Source: Rev Cardiovasc Med. 2025 Apr 27;26(4):27734. doi: 10.31083/RCM27734 (PMC12059729; doi:10.31083/RCM27734)

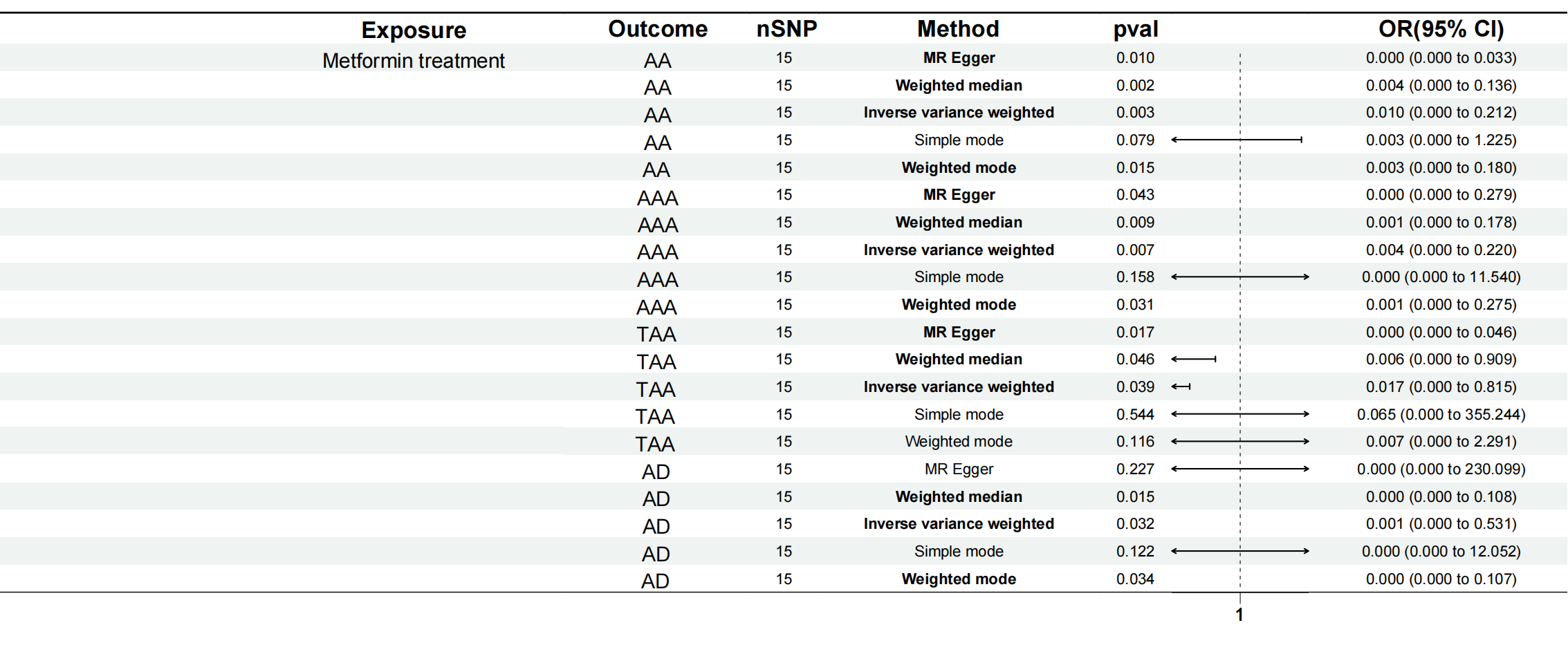

Supplement: Supplementary file 1 [file 2153-8174-26-4-27734-s1.zip › Figure S1.tif]
